# New solid phase methodology for the synthesis of biscoumarin derivatives: experimental and in silico approaches

**DOI:** 10.1186/s13065-022-00844-8

**Published:** 2022-07-11

**Authors:** Elham Zarenezhad, Mohammad Nazari Montazer, Masoumeh Tabatabaee, Cambyz Irajie, Aida Iraji

**Affiliations:** 1 Noncommunicable Diseases Research Center, Fasa University of Medical Sciences, Fasa, Iran; 2 Department of Medicinal Chemistry, Faculty of Pharmacy, Tehran University of Medical Sciences, Tehran, Iran; 3 Department of Chemistry, Yazd Branch, Islamic Azad University, Yazd, Iran; 4 Department of Medical Biotechnology, School of Advanced Medical Sciences and Technologies, Shiraz University of Medical Sciences, Shiraz, Iran; 5 Stem Cells Technology Research Center, Shiraz University of Medical Sciences, Shiraz, Iran; 6 Central Research Laboratory, Shiraz University of Medical Sciences, Shiraz, Iran

**Keywords:** Molecular dynamics simulations, MM-GBSA, Biscoumarin, MoO_3_-nanoparticle

## Abstract

The simple and greener one-pot approach for the synthesis of biscoumarin derivatives using catalytic amounts of nano-MoO_3_ catalyst under mortar-pestle grinding was described. The use of non-toxic and mild catalyst, cost-effectiveness, ordinary grinding, and good to the excellent yield of the final product makes this procedure a more attractive pathway for the synthesis of biologically remarkable pharmacophores. Accordingly, biscoumarin derivatives were successfully extended in the developed protocols*.* Next, a computational investigation was performed to identify the potential biological targets of this set of compounds. In this case, first, a similarity search on different virtual libraries was performed to find an ideal biological target for these derivatives. Results showed that the synthesized derivatives can be α-glucosidase inhibitors. In another step, molecular docking studies were carried out against human lysosomal acid-alpha-glucosidase (PDB ID: 5NN8) to determine the detailed binding modes and critical interactions with the proposed target. In silico assessments showed the gold score value in the range of 17.56 to 29.49. Additionally, molecular dynamic simulations and the MM-GBSA method of the most active derivative against α-glucosidase were conducted to study the behavior of selected compounds in the biological system. Ligand 1 stabilized after around 30 ns and participated in various interactions with Trp481, Asp518, Asp616, His674, Phe649, and Leu677 residues.

## Introduction

**Fig. 1:**
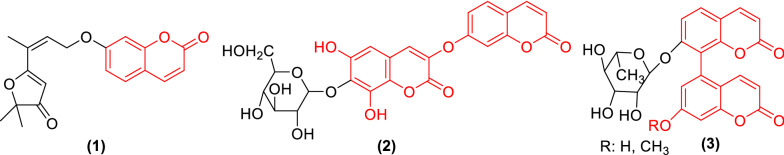
Natural coumarin compounds

Coumarin compounds exhibited brilliant and remarkable pharmaceutical activities [1]. Geiparvarin (**1**) [2], daphsafnin (**2**) [3] as well as daphjamilin (**3**) [4] known as natural coumarin compounds with anti-cancer and anti-monoamine oxidase activities (Fig. 1).

**Fig. 2:**
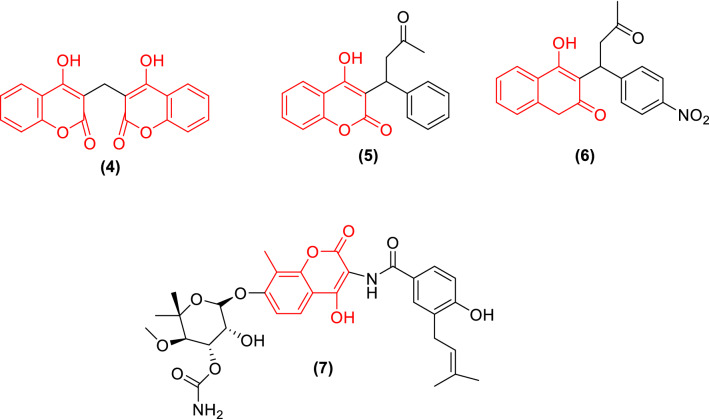
coumarin derivatives drugs

Biscoumarins derivatives comprise a diverse and interesting group of heterocyclic drugs which are extremely important for their biological activities. Some coumarin derivatives, in general, and biscoumarins, in particular, are well known for their biological activity [5]. Some approved drugs with various properties are shown in Fig. 2. Dicoumarol (**4**) and warfarin (**5**) are naturally anticoagulant that depletes stores of vitamin K. Another anticoagulant drug is acenocoumarol (**6**) which inhibited the reduction of vitamin K by vitamin K reductase [6]. Aminocoumarin (**7**) as a coumarin derivative was an antibiotic for the treatment of *staphylococci* infections.

Several methods have been reported for the synthesis of biscoumarins, recently Kurt et al. synthesized a series of novel bis-coumarin derivatives containing triazole moiety [7]. Bavandi et al. demonstrated the synthesis of bis-4-hydroxy coumarins derivatives in the presence of *Porcine pancreas* lipase as a green catalyst [8]. Zeynizadeh et al. reported the novel magnetic graphene oxide/Zn–Ni–Fe layered double hydroxide nanocomposite for the green preparation of biscoumarins [9]. Hagiwara et al. showed the use of Et_2_AlCl as a Lewis acid for the condensation of 4-hydroxycoumarin and aldehydes in acetonitrile or dichloromethane at room temperature [10]. In continue, other researchers reported a similar reaction using piperidine, molecular iodine [11], tetrabutylammonium bromide (TBAB) [12], heteropolyacids [13], phospho- tungstic acid [14] and sodium dodecyl sulfate (SDS)[15] as catalysts. Some of these procedures required refluxing for hours in organic solvents, use of expensive catalysts, and tedious work-up. With the increasing public concern over environmental degradation, water is commonly considered as a benign solvent because of its non-toxicity, lower cost, and abundant natural occurrence [16].

Recently, mechanochemical synthesis has received lots of attention among synthetic chemists as a brilliant standard technology [17]. Many chemical reactions such as Grignard reactions [18] reductions [19] click reactions [20] and Knoevenagel condensations [21] have been carried out using the green and mechanochemical techniques. The importance of this type of protocol is free from organic solvents.

Metal oxide nanoparticles possess huge surface areas as a useful heterogeneous catalyst traditionally for catalyzing organic reactions. They are defined as friendly, and environmentally materials with attractive physical and chemical properties [22]. Metal oxide nanoparticles are effective catalysts that can replace nonselective, unstable, or toxic catalysts [23].

MoO_3_-nanoparticle and its derivatives are extensively attractive due to its unique structure, it was used as an inexpensive, eco-friendly, and highly reactive catalyst. Moreover, this non-toxic catalyst can be easily applied for diverse organic synthesis, generating corresponding products in excellent yields [24]. MoO_3_ showed significant Lewis acid property for diverse organic transformations in the liquid phase [25]. It demonstrated quite active over a wide range of temperatures and resistant to thermal excursions [26].

Similarity search, a subcategory of ligand-based virtual screening, has emerged as a reliable, fast, and inexpensive method that finds compounds with high similarity in some ways, especially molecular features, to known bioactive molecules. The assumption that molecules that are globally similar in structure could exhibit similar biological activity is generally valid [27, 28]. Once a target has been identified, molecular docking and molecular dynamic simulation, known as structure-based virtual screening, can be applied [29–31]. In docking assessments databases of available molecules are docked into the region of interest of protein in silico and scored based on predicted interactions with the site. In these cases, compounds with a higher affinity toward the target can be discovered [32]. Molecular dynamic (MD) simulation is an effective technique to measure the behavior of fluids at the molecular level. In the MD simulation, the movement of system particles is determined in a certain period and the system evolution is investigated. MD simulation is a process similar to real experiments. Thus, it is an ideal tool to study the different interactions and conformation of ligands in the biological system at the nanometer scale [33, 34].

As a result, with inspiration from the remarkable biological activities of biscoumarin derivatives [35–37] hereby, the solid-phase pathway for Knoevenagel–Michael reactions of biscoumarins (3) by the reaction of aldehydes (2) and 4- hydroxycoumarin (1) in the presence of 10 mol% nano-MoO_3_ were reported. The products were achieved by grinding the reactants in a mortar with a pestle under solvent*-*free conditions. Similarity search analysis and molecular docking studies were performed to find an ideal biological target for this set of compounds. Finally, MD simulations were combined with the generalized-Born surface area method (MM/GBSA) to evaluate the solvation free energy of the protein and the ligand in the dynamic situations.

## Experimental section

### General

Solvents were purified by standard procedures. Reactions were followed by TLC using. Melting points were determined with an Electrothermal 9100 apparatus in open capillary tubes and are uncorrected. The chemicals used in this work were purchased from Fluka or Merck companies and were used without further purification. FT-IR spectra were recorded on Thermo Nicolet Nexus 670 and ^1^H NMR were determined by Bruker Avance 300 MHz spectrometers.

#### General procedure for the synthesis of biscoumarin derivatives using mortar–pestle grinding

An aromatic aldehyde (1 mmol), 4-hydroxycoumarin (2 mmol) and MoO_3_ (10 mol%) were subjected to mortar and pestle grinding for specified time. After completion of the reaction (monitored by TLC), 3 mL of distilled water was added to the reaction mixture and the product was extracted and washed with water (10 ml). Recrystallization from hot ethanol afforded pure biscumarin derivatives in good to excellent yield. The structure of all products was confirmed by appropriate spectroscopic and physical methods (Melting point or IR, ^1^HNMR) with those reported or with authentic samples prepared by the conventional method.

#### The procedure for the synthesis of 3, 3'-((4-hydroxyphenyl) methylene) bis (4-hydroxy-2H-chromen-2-one) using reflux:

4*-*hydroxybenzaldehyde (1 mmol) and 4-hydroxycoumarin (2 mmol) were taken in 25 mL round bottom flask followed by the addition of a different solvent (15 mL) and MoO_3_ (10 mol%) was stirred and reflux for the appropriate times. The progress of the reaction was monitored by TLC, the reaction mixture was washed with H_2_O (10 mL) and EtOAc (10 mL) to almost afford the pure product. The crude products was recrystallized from hot ethanol to obtain the pure compound.

*3,3'-((4-chlorophenyl)methylene)bis(4-hydroxy-2H-chromen-2-one)* (***3h***): FT-IR (KBr) (KBr, ν cm^−1^) 3072, 2923,2856, 1667, 1610, 1562, 1349, 1095, 764; ^1^H NMR (300 MHz, CDCl_3_) δ (ppm): 11.5 (s, 1H, OH), 11.3 (s, 1H, OH), 8.0 (s, 2H, arom), 7.6 (s, 2H, arom),7.1–7.4 (m, 8H, arom), 6.04 ( s, 1H, CH).

*3,3'-((4-methoxyphenyl)methylene)bis(4-hydroxy-2H-chromen-2-one) (****3i****):* FT-IR (KBr, cm^−1^) 3065, 2989, 2842, 2727, 1665, 1613, 1563, 1349, 1042, 769; ^1^H NMR (300 MHz, CDCl_3_) δ (ppm): 11.52 (s, 1H, OH), 11.31 (s, 1H, OH), 8.04 (s, 2H, arom), 7.65–7.63 (m, 2H, arom),7.42–7.39 (m, 4H, arom),7.27 (s,2H, arom) 7.14- 7.12 (m, 2H, arom), 6.04 ( s, 1H, CH), 3.89 (s, 3H, CH_3_).

*3,3'-((4-nitrophenyl)methylene)bis(4-hydroxy-2H-chromen-2-one) (****3j****):* FT-IR (KBr, ν cm^−1^) 3064, 2922, 2857, 2720, 1654, 1609, 1564, 1339, 1096, 764 ^1^H NMR (300 MHz, CDCl_3_) δ (ppm): 11.56 (s, 2H, OH), 8.15–8.02 (m, 4H, arom), 7.65–7.26 (m, 8H, arom), 6.11 ( s, 1H, CH).

*3,3'-((3-nitrophenyl)methylene)bis(4-hydroxy-2H-chromen-2-one) (****3k****):* FT-IR (KBr, ν cm^−1^) 3071, 2925, 2859, 2772, 1656, 1609, 1526, 1346, 1010, 763; ^1^H NMR (300 MHz, CDCl_3_) δ (ppm): 11.58 (s, 1H, OH), 11.38 (s, 1H, OH), 8.14–7.99 (m, 4H, arom), 7.67–7.43 (m, 8H, arom),6.14 (s, 1H, CH).

*3,3'-((2,4-dichlorophenyl)methylene)bis(4-hydroxy-2H-chromen-2-one)(****3l****):* FT-IR (KBr, ν cm^−1^) 3071, 2925, 2861, 1653, 1311, 1096, 756; ^1^H NMR (300 MHz, CDCl_3_) δ (ppm): 11.60 (s, 1H, OH), 11.59 (s, 1H, OH), 7.98–7.34 (m, 11H, arom), 6.07–6.01 (s, 1H, CH).

*3,3'-(phenylmethylene)bis(4-hydroxy-2H-chromen-2-one) (****3m****):* FT-IR (KBr, ν cm^−1^) 3065,3000, 2927, 2737, 1659, 1609, 1336, 1090, 755; ^1^H NMR (300 MHz, CDCl_3_) δ (ppm): 11.43 (s, 2H, OH), 8.04 (s, 2H, arom), 7.39–7.31 (m, 9H, arom), 6.10 (s, 1H, CH).

*4-(bis(4-hydroxy-2-oxo-2H-chromen-3-yl)methyl)benzaldehyde (****3n****):* FT-IR (KBr, ν cm^−1^) 3068, 2923, 2806, 2730, 1701, 1607, 1097, 764, ^1^H NMR (300 MHz, CDCl_3_) δ (ppm): 11.54 (s, 1H, OH), 11.34 (s, 1H, OH), 9.93 (s, 1H, arom), 7.97–7.38 (m, 12H, arom), 6.10 (s, 1H, CH).

### Insights into the biological activities of biscoumarin derivatives

In order to propose biological activities of the synthesized compounds, three steps computational process was applied, using ligand-based similarity search, molecular docking, and molecular dynamics.

#### Similarity-based analog searching

To find an ideal biological target for this set of compounds, ligand-based similarity search on several libraries including SwissTargetPrediction (http://www.swisstargetprediction.ch/), PubChem similarity search (https://pubchem.ncbi.nlm.nih.gov/), SEA Search Server (https://sea.bkslab.org/), MolTarPred (https://moltarpred.marseille.inserm.fr/), SuperPred was performed. In these databases, the structure of compounds was uploaded and different searching approaches including fingerprint and shape-based similarity pharmacophores were applied automatically to find the most similar bioactive agents compared to the synthesized compounds.

#### Procedure for docking studies

In this study, the Gold docking program was used to carry out the docking calculations between the biscoumarin analogs and the binding site of human lysosomal acid-alpha-glucosidase (PDB ID: 5NN8, https://www.rcsb.org/structure/5nn8) [38]. The protein structure was prepared using the Discovery Studio Client so that ligands and waters were removed from 5NN8 and all hydrogens were added. The box for docking calculations was built taking into account the center of the co-crystallized ligand for the enzyme with a 10 Å radius sphere around the co-crystallized ligand.

First, GOLD docking program with different functions was used for docking analyses via re-dock acarbose inside the 5NN8. All other options were set as default. The best accuracy with the lowest RMSD was seen in the ChemScore fitness function. The 3D structures of ligands were first generated by the Hyperchem and then energy minimization and optimization were performed to generate the initial confirmation via molecular mechanics (Amber) followed by molecular dynamic (AM1) approaches. These approaches are widely applied in ligand-docking and MD simulations which offer acceptable criteria to determine the proper and favorable molecular arrangement and potential energy of a molecule [39–41]. The derivatives were docked into the active site of protein using default parameters 10 runs for each ligand; Genetic algorithm with 100% efficacy, min ops 10,000; max ops 125,000 were chosen, All other options were set as default. The top ChemScore value was used for further analysis. The higher value confirms better interaction with the active site. Finally, protein–ligand interactions were analyzed with Discovery Studio Visualizer [42–44].

#### Molecular dynamics simulations

Starting model was obtained by imposing the best ChemScore to acid-α-glucosidase (PDB ID: 5NN8). MD simulations were conducted using the desmond operator of Schrodingers suit maestro [45–47]. To build the system for MD simulation, the protein–ligand complexes were solvated with SPC explicit 22,530 water molecules and placed in the center of an orthorhombic box in the periodic boundary condition. The system’s charge was neutralized by adding 84 atoms of Na and 63 atoms of Cl to simulate the real cellular ionic concentrations, respectively. The MD simulations protocol involved minimization, pre-production, and finally production MD simulation steps. In the minimization procedure, the entire system was allowed to relax for 2500 steps by the steepest descent approach. Then the temperature of the system was raised from 0 to 300 K with a small force constant on the enzyme to restrict any drastic changes. MD simulations were performed via NPT (constant number of atoms; constant pressure, i.e., 1.01325 bar; and constant temperature, i.e., 300 K) ensemble. The Nose–Hoover chain method was used as the default thermostat with 1.0 ps interval and Martyna-Tobias-Klein as the default barostat with 2.0 ps interval by applying an isotropic coupling style. Long-range electrostatic forces were calculated based on the particle-mesh-based Ewald approach with the cutoff radius for Columbia forces set to 9.0 Å. Finally, the system was subjected to produce MD simulations for 100 ns for each protein–ligand complex. During the simulation, every 1000 ps of the actual frame was stored. The dynamic behavior and structural changes of the systems were analyzed by the calculation of the RMSD and RMSF. Subsequently, the energy-minimized structure calculated from the equilibrated trajectory system was evaluated for the investigation of each ligand–protein complex interaction.

## Results and discussion

### Chemical synthesis

**Fig. 3:**
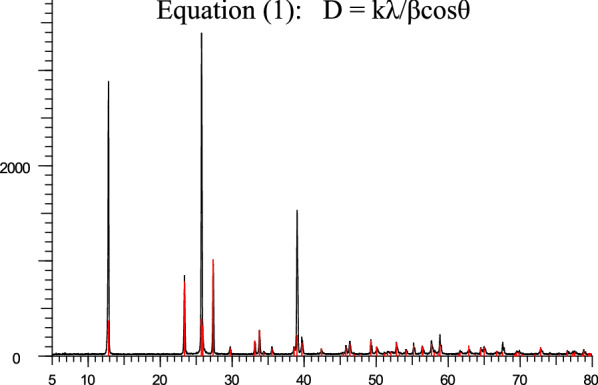
PXRD patterns of the synthesized MoO_3_ nano-crystals

**Fig. 4:**
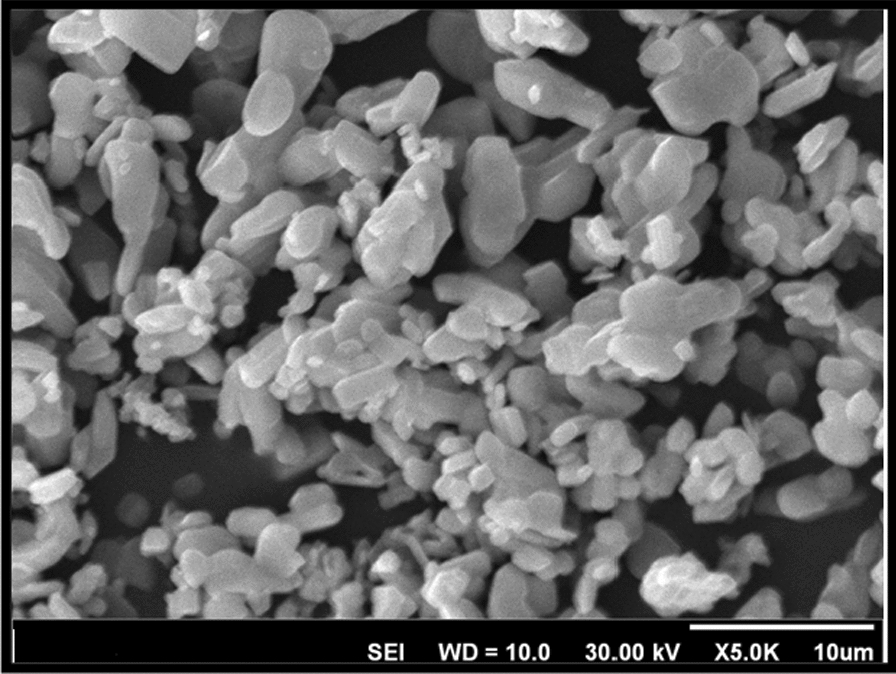
Scanning electron micrographs for prepared nano-MoO_3_

MoO_3_-nanoparticles were previously synthesized [26, 48]. For identification of nanostructure powder X-ray diffraction (PXRD) and scanning electron microscopy (SEM) analysis were carried out. Figure 3, displayed powder X-ray diffraction (PXRD), and the surface morphology and the diameter of the nanostructure were studied by SEM in Fig. 4. Debye–Scherrer Eq. (1) was used for calculating crystal size structure, according to this equation: D is the mean size of crystalline, k is a constant (= 0.9 assuming that the particles are spherical), λ is the X-ray wavelength, β is the line width (obtained after correction for the instrumental broadening) and θ is the angle of diffraction (Bragg angle). The average particle size obtained from XRD data is approximately ~ 50 nm [26]

1 $${\text{D }} = {\text{ k}}\lambda /\beta {\text{cos}}\theta$$

**Fig. 5:**
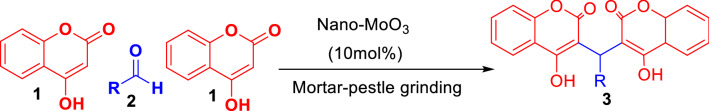
General pathway for synthesis of biscoumarin derivatives

The synthetic route to access compounds **3a- 3n** are achieved by the solid phase general pathway illustrated in Fig. 5. The condensation reaction between 4-hydroxycoumarin and various aldehydes is summarized. This solvent-free reaction needed only a few minutes of reaction time. This kind of reaction is expected to be the most economical method since neither solvent is used.

### Effect of different catalyst

The amount of catalyst has shown an excellent effect on the rate and yield of the reaction, To optimize the reaction conditions, the amount of various catalysts were applied in the solid phase synthesis of 3,3'-((4-hydroxphenyl) methylene)bis(4-hydroxy-2H-chromen-2-one) 3a from the condensation of 4-hydroxycoumarin with 4*-*hydroxybenzaldehyde and the best result at room temperature was obtained (Table 1).

**Table 1:** Optimization of the solvent-free reaction

Entry	Catalyst (mol%)	Time (min)	Yield (%)^a^
1	**-**	60	5
2	H_2_SO_4_ (15)	35	60
3	CF_3_CO_2_H(10)	50	40
4	CCl_3_CO_2_H (10)	50	55
5	PTSA(10)	50	45
6	AlCl_3_(10)	50	60
7	MoO_3_(10)	20	93
8	MoO_3_(11)	20	93
9	MoO_3_(12)	20	93
10	MoO_3_(8)	20	75
11	MoO_3_(4)	20	70

^a^Isolated yield

According to the result in Table 1, when the reaction was performed in the absence of catalyst, longer reaction time was required (60 min) and very low yield of product was achieved (≤ 5%) even if the reaction time was prolonged (Table 1, entry 1). To obtain the satisfactory results of (3a), the reaction using various homogeneous and heterogeneous Bronsted and/or Lewis acids was performed (Table 1, entries 2–7). Accordingly, the nano-MoO_3_ was the best catalyst and applied for all reactions. Using 10 mol % of nano-MoO_3_, **3a** was isolated in 93% yield after the progress of the reaction for a few minutes (Table 1, entry7). By increasing the amount of the catalyst in the model reaction, no change was observed in the time and yield of the reaction (Table 1, entries 8–9). The decline in the use of the catalyst amount less than 10% resulted in low yields (Table 1, entries 10–11).

Under the optimized reaction conditions, a series of biscoumarins derivatives (**3a–n**) were synthesized, the structure of all products was confirmed by appropriate spectroscopic and physical methods (Table 2).

**Table 2:**
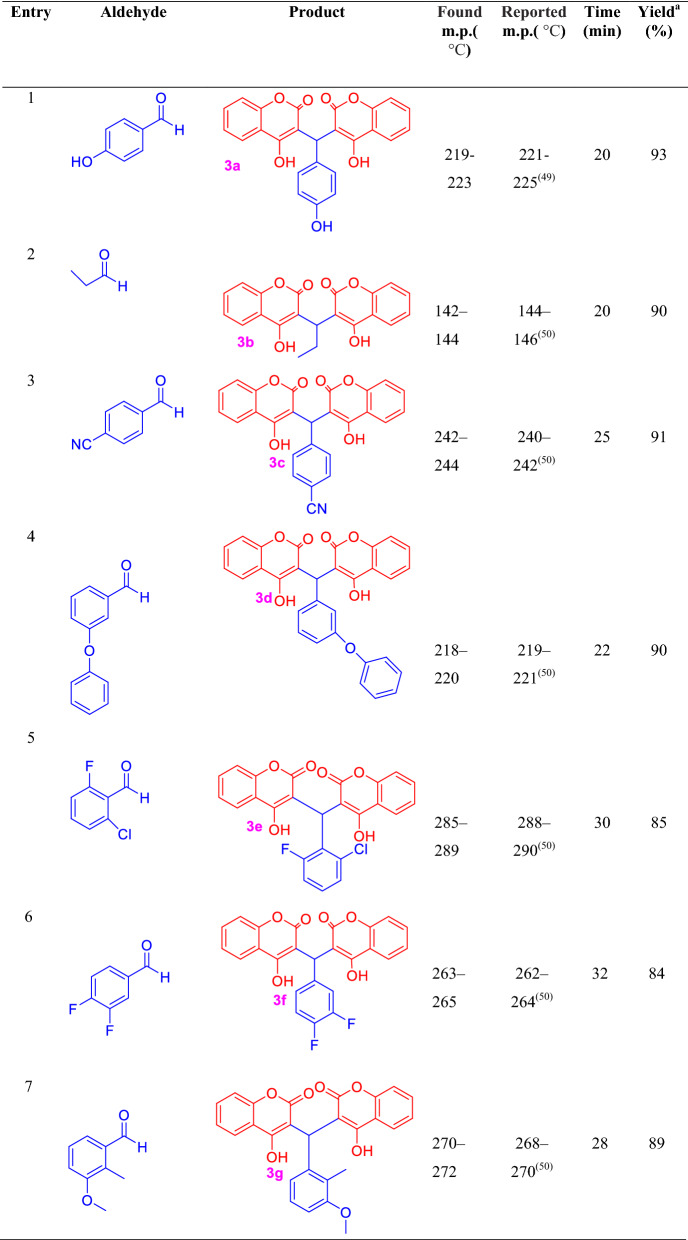

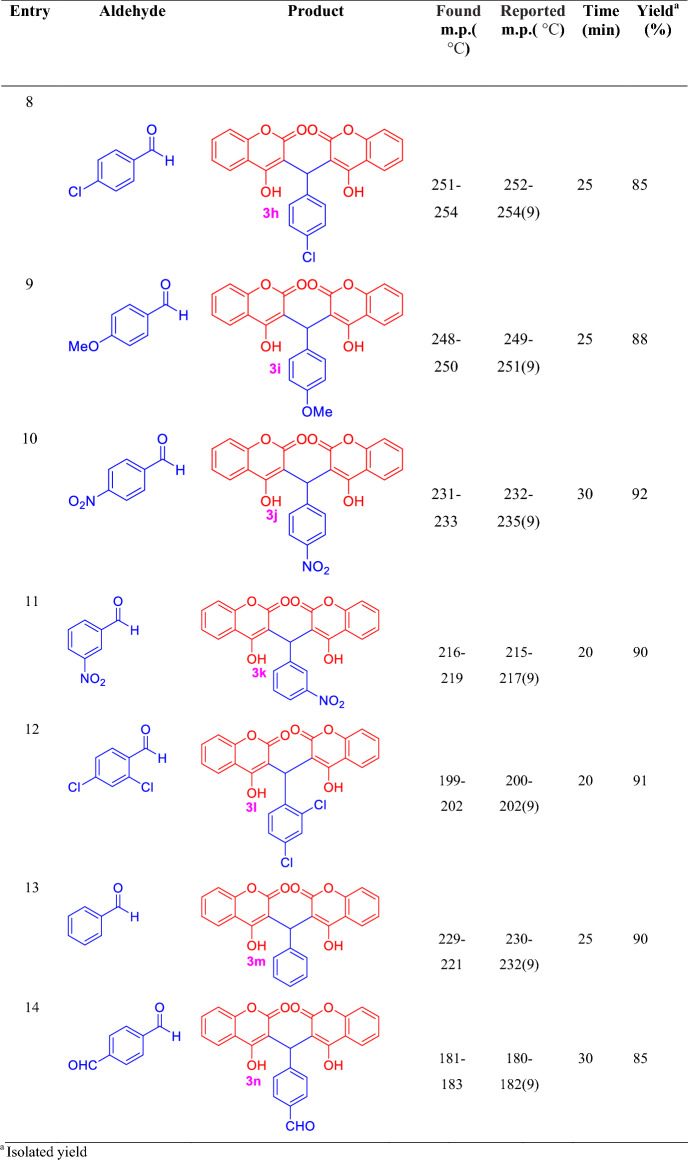
Synthesis of biscoumarins derivatives (4a–n) using nano-MoO_3_ under mortar and pestle grinding

^a^Isolated yield

### Effect of different solvents and temperature in reflux condition

Our next approach was to study various solvents in different temperatures on the model reaction of 4-hydroxycoumarin and 4*-*hydroxybenzaldehyde in the presence of (10 mol %) of MoO_3_ for preparation of **3a** (Table 3).

**Table 3:** Optimization of the solvent and temperature in room temperature or reflux condition

Entry	Solvent	T (°C)	Time (min)	Yield (%)^a^
1	CH_3_CN	r.t	120	30
2	CH_3_CN	Reflux	120	50
3	CH_2_Cl_2_	r.t	120	40
4	CH_2_Cl_2_	Reflux	120	55
5	EtOH	r.t	120	60
6	EtOH	Reflux	120	68
7	H_2_O	r.t	120	70
8	H_2_O	Reflux	120	77
9	EtOH/H_2_O	r.t	120	80
10	EtOH/H_2_O	Reflux	120	91
11	MeOH	r.t	120	70
12	MeOH	Reflux	120	72

^a^Isolated yield

The model reaction was performed in the different solvents and various temperatures (Table 3 entries 1–12), the results confirmed that carrying out the reaction in EtOH/ H_2_O (1:1) in reflux condition gave the highest yield (Table 3, entries 10). Eventually, the reaction using mortar–pestle grinding method is low cost, more efficient, and simple reaction without the usage of organic solvent that gives the desired product.

### Effect of different aldehydes

After reaction optimization, different aldehydes were chosen and reacted under optimum conditions. This multi-component and solid phase approach can be used for both aromatic aldehydes with electron-withdrawing and electron-donating groups. Furthermore, a wide range of aromatic aldehydes was successfully used in this reaction with excellent results.

**Fig. 6:**
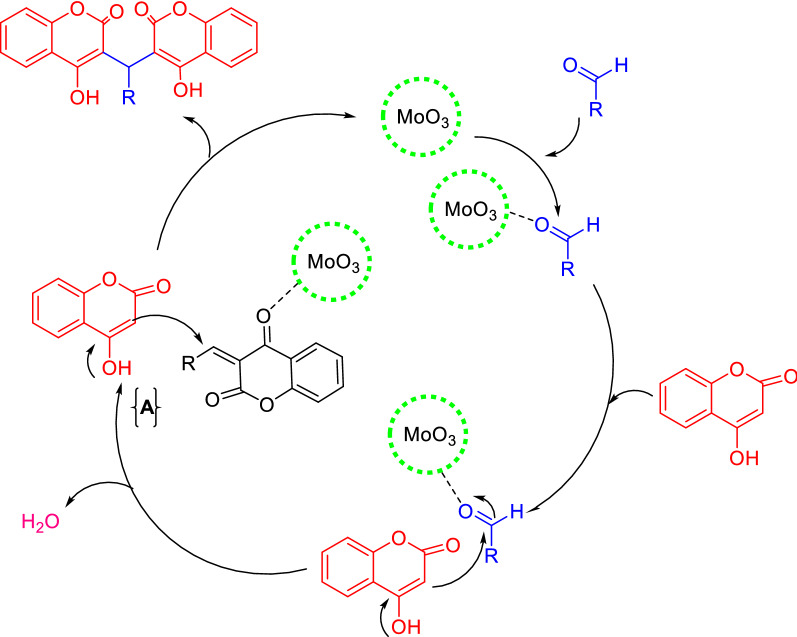
A plausible mechanism for synthesis of biscoumarins derivatives using MoO_3_

A proposed mechanistic route for the condensation of aldehydes and 4-hydroxycoumarin that rationalizes the formation of the products is exhibited in (Fig. 6). First, nucleophilic attack of 4-hydroxycoumarin to the activated aldehyde (by MoO_3_ coordination), followed by H_2_O elimination provides intermediate “A” that was further activated by MoO_3_. This, in turn, undergoes a second nucleophilic attack by another 4-hydroxycoumarin to provide the final product.

### Similarity search and docking studies

The similarity metrics analysis on several libraries indicated that the synthetic biscoumarin derivatives can be ideal α-glucosidase inhibitors. Regarding the similarity of reported α-glucosidase inhibitors with the designed structure, molecular docking evaluations were performed to study the binding mode of all derivatives with the α-glucosidase active site. α-glucosidase enzyme catalyzes the hydrolysis of starch to simple sugars which leads to an increase in blood glucose levels. α-glucosidase inhibitors can be ideal and effective anti-diabetic agents [51–53]. Docking studies of the mentioned compounds were carried out with gold docking software using different score fitness functions including chem score, gold score, ChemPLP, and the best accuracy with the lowest RMSD was seen in the ChemScore fitness function. Validation of the molecular docking method was done by redocking the crystallographic ligand of the target enzyme, gainst 5NN8, which testified the validation of the docking calculations. Alignment of the best pose of acarbose in the active site of α-glucosidase and crystallographic ligand recorded an RMSD value of 1.45 Å via ChemScore fitness functions. Results of ChemScore fitness values were reported in Table 4. It should be noted that fitness scores in gold software are dimensionless and the higher scale value showed better interactions with the active site [54, 55].

**Table 4:** Docking scores and interactions of compounds against α-glucosidase

Compound	ChemScore value	Interactions with key residue
1	29.49	Asp404, Phe525, Arg600, Phe649, Leu650, Leu678
2	23.05	Trp376, Trp481, Leu650, Leu677, Leu678
3	17.56	Ala284, Trp376, Trp618, Phe649, Leu650
4	25.51	Trp376, Asp404, Trp481, Asp518, Met519, Phe525, Asp616, Phe649, Leu650, Leu677, Leu678
5	18.54	Trp481, Leu650, Leu677, Leu678
6	20.35	Trp376, Trp481, Phe649, Leu650, Leu678
7	22.92	Trp481, Phe649, Leu650, Ser676, Leu677, Leu678
8	24.63	Trp376, Trp481, Phe649, Leu650, Leu677
9	23.41	Trp376, Phe649, Leu650, Leu678
10	24.05	Asp282, Ala284, Ala555, Asp616, Trp618, Leu650
11	18.74	Trp376, Phe649, Leu650
12	24.06	Trp376, Trp481, Phe649, Leu650, Ser676, Leu677, Leu678
13	21.89	Trp376, Arg411, Trp481, Phe649, Leu650 Leu677, Leu678
14	22.26	Trp376, Trp481, Phe649, Leu650, Ser676, Leu677, Leu678

**Fig. 7:**
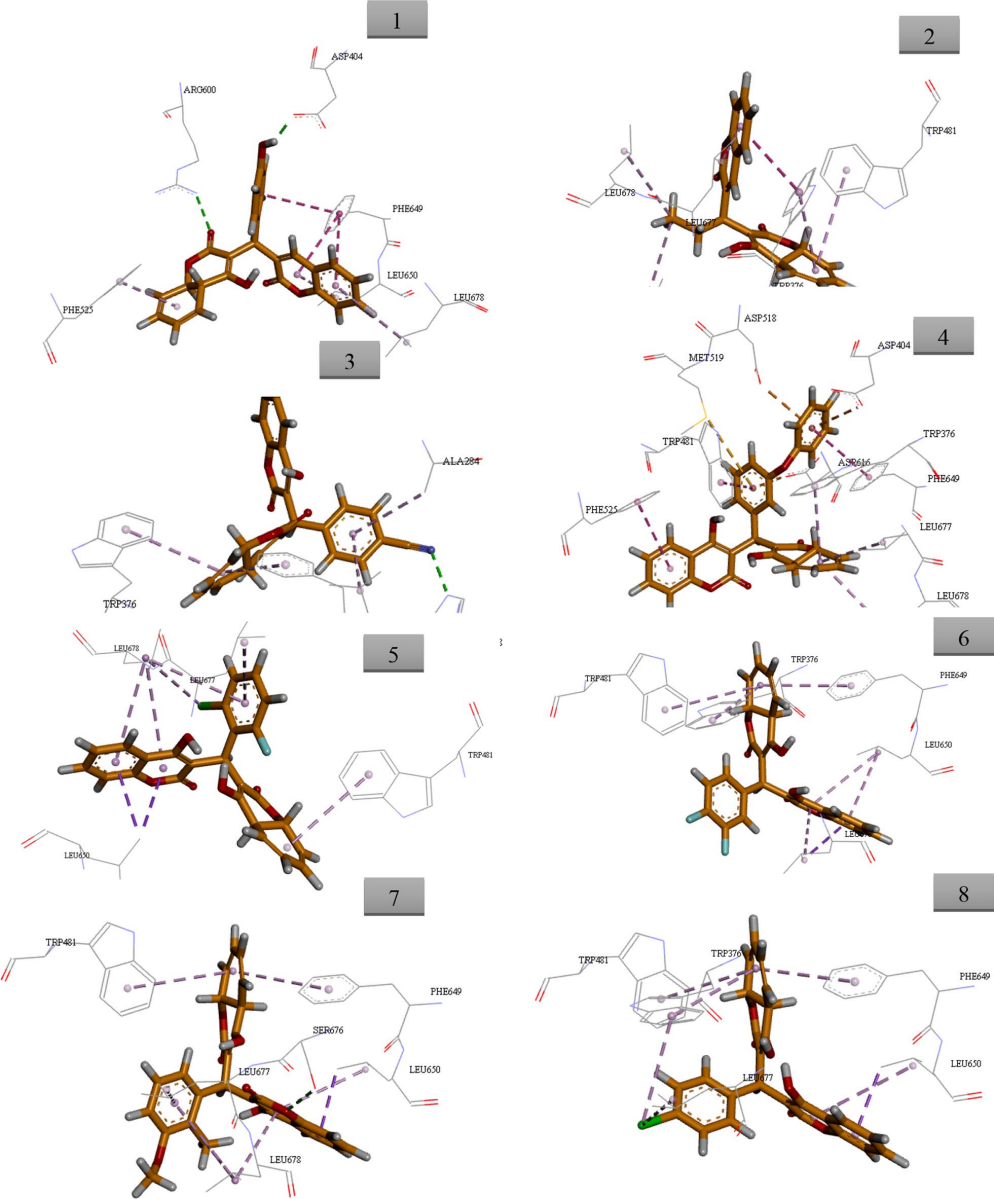

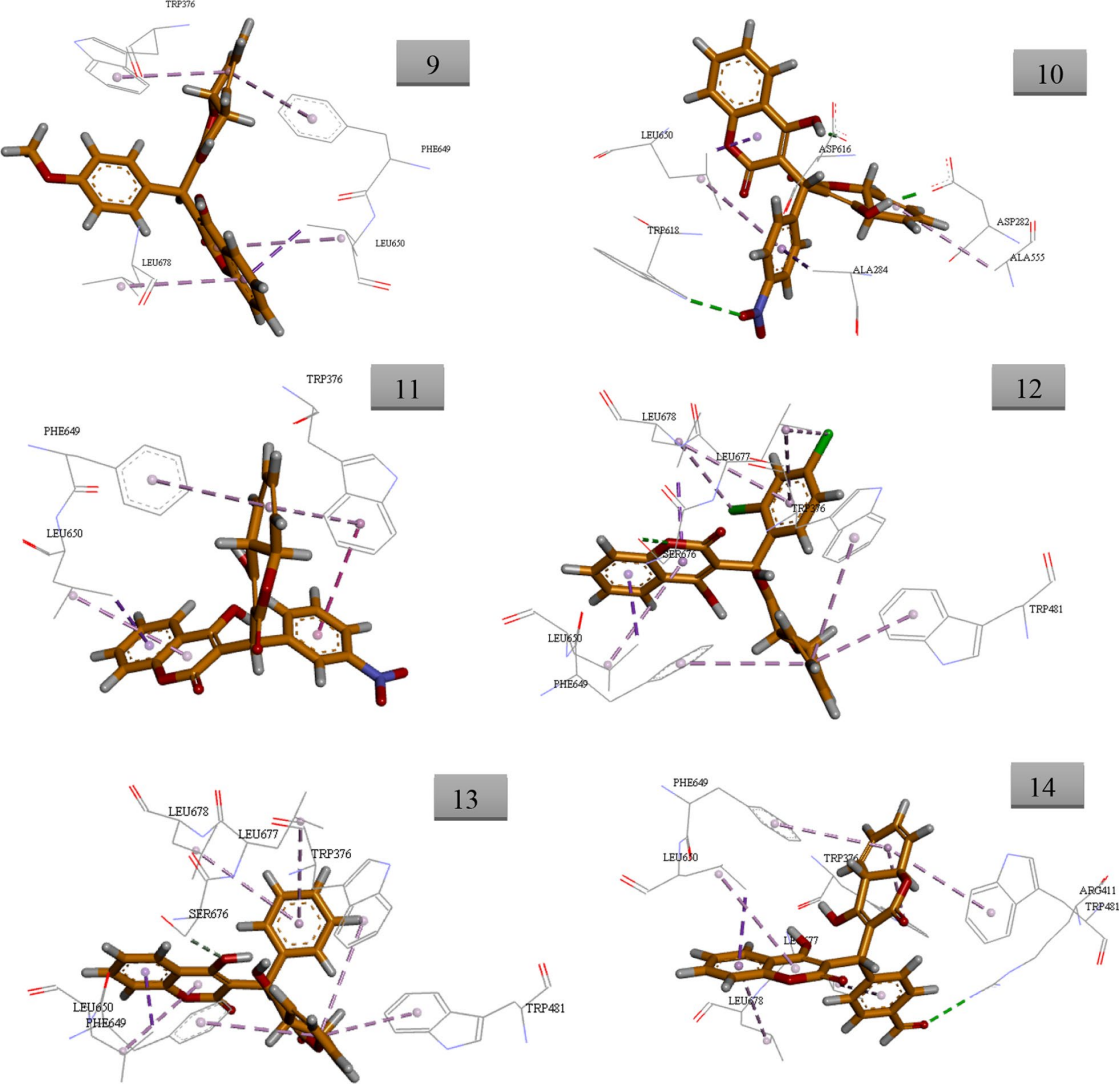
Docking conformations of compounds 1–14 (orange stick) in the α-glucosidase active site. Hydrogen bonds are depicted in green dashed lines, π-π stacked interactions are depicted in dark pink dashed lines, π-aryl interactions are depicted in pink dashed lines pi-sulfur interactions are depicted in pale orange dashed lines and and pi-anion interactions are depicted in dark orange dashed lines

The predicted binding pose of top-ranked docked compounds was presented in Fig. 7. All the residues involved in molecular interaction are shown in stick form and colored by atom types in which carbon is depicted in orange and oxygen in red

Images were created by Discovery Studio Visualizer v20.1.0.19295.

As can be seen in Fig. 7, compound 1 demonstrated the best docking score (29.49) compared to other derivatives. The chromen-2-one moiety of compound 1 made two pi-alkyl interactions with Leu650 and Leu678 as well as two pi-pi T-shaped interactions with Phe649. On the other side of the molecule, 4-hydroxy chromen-2-one showed a hydrogen bond with Arg600 plus a pi–alkyl interaction with Phe525. Another H-bounding interaction was also seen between the oxygen of 4-hydroxyphenyl ring with Asp404. 4-hydroxyphenyl also recorded additional pi-pi stacked interaction with Phe649.

### MD simulations

**Fig. 8:**
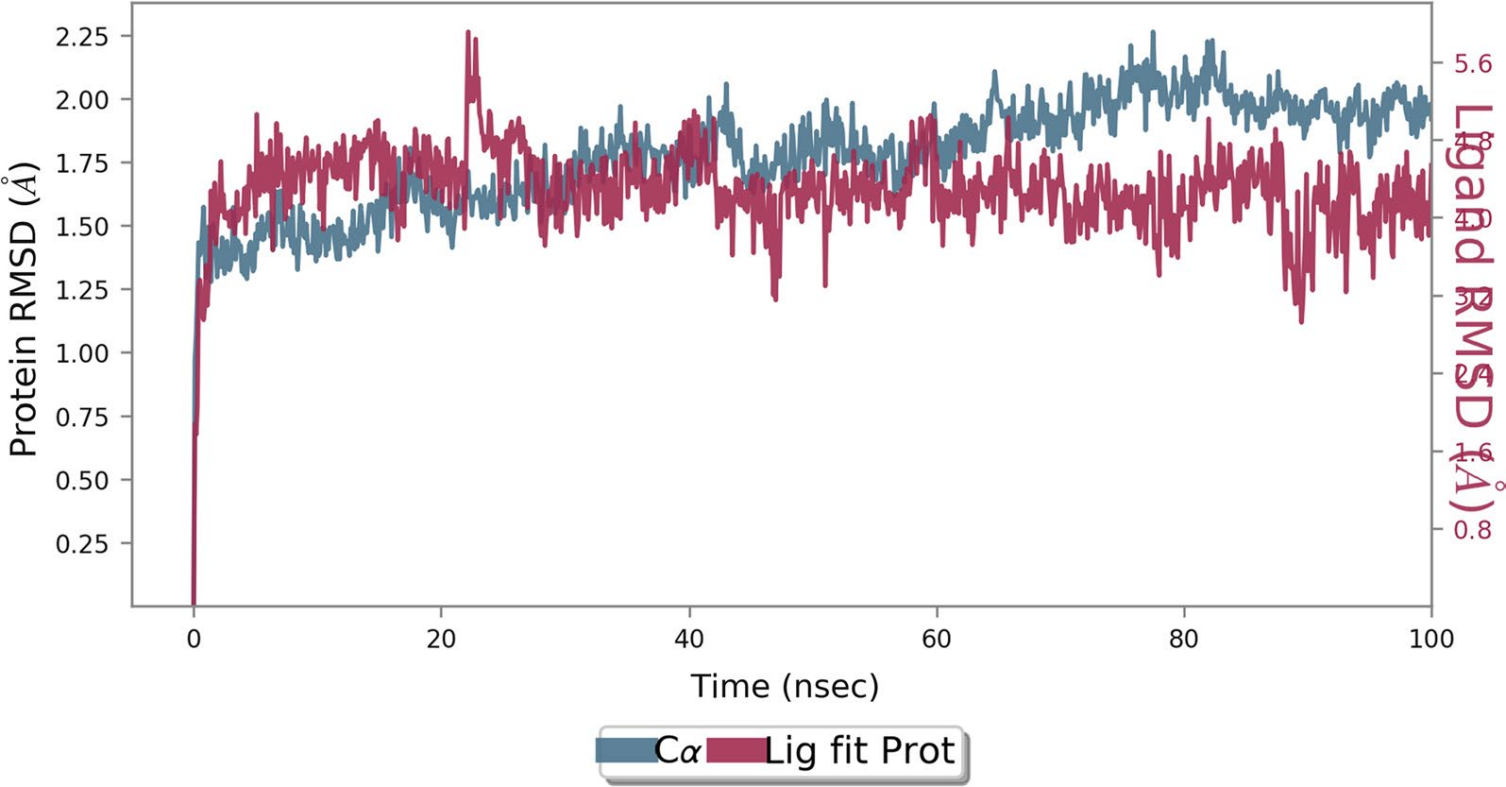
RMSD plot of the enzyme in complexed compound 1 in the MD simulation time. RMSD values of the Ca atoms of the protein are depicted in blue, and ligand-complex values are exhibited in red

Molecular docking studies alone can be misleading, as they are performed in static conditions. To validate these results and better understand the potential for interaction of ligand 1 and their ability to coordinate with α-glucosidase active site MD simulations were performed. To study the steadiness of the protein–ligand complex, the root mean square deviation (RMSD) of the complexed backbone was investigated in MD simulation. As can be seen in Fig. 8, the simulation period was adequate to reach a balanced ligand-complex structure over the simulation time and the RMSD values stabilize around a fixed value of 1.60 Å. Changes of the order of 1–3 Å are perfectly acceptable for small, globular proteins. Changes much larger indicate the protein is undergoing a large conformational change during the simulation.

**Fig. 9:**
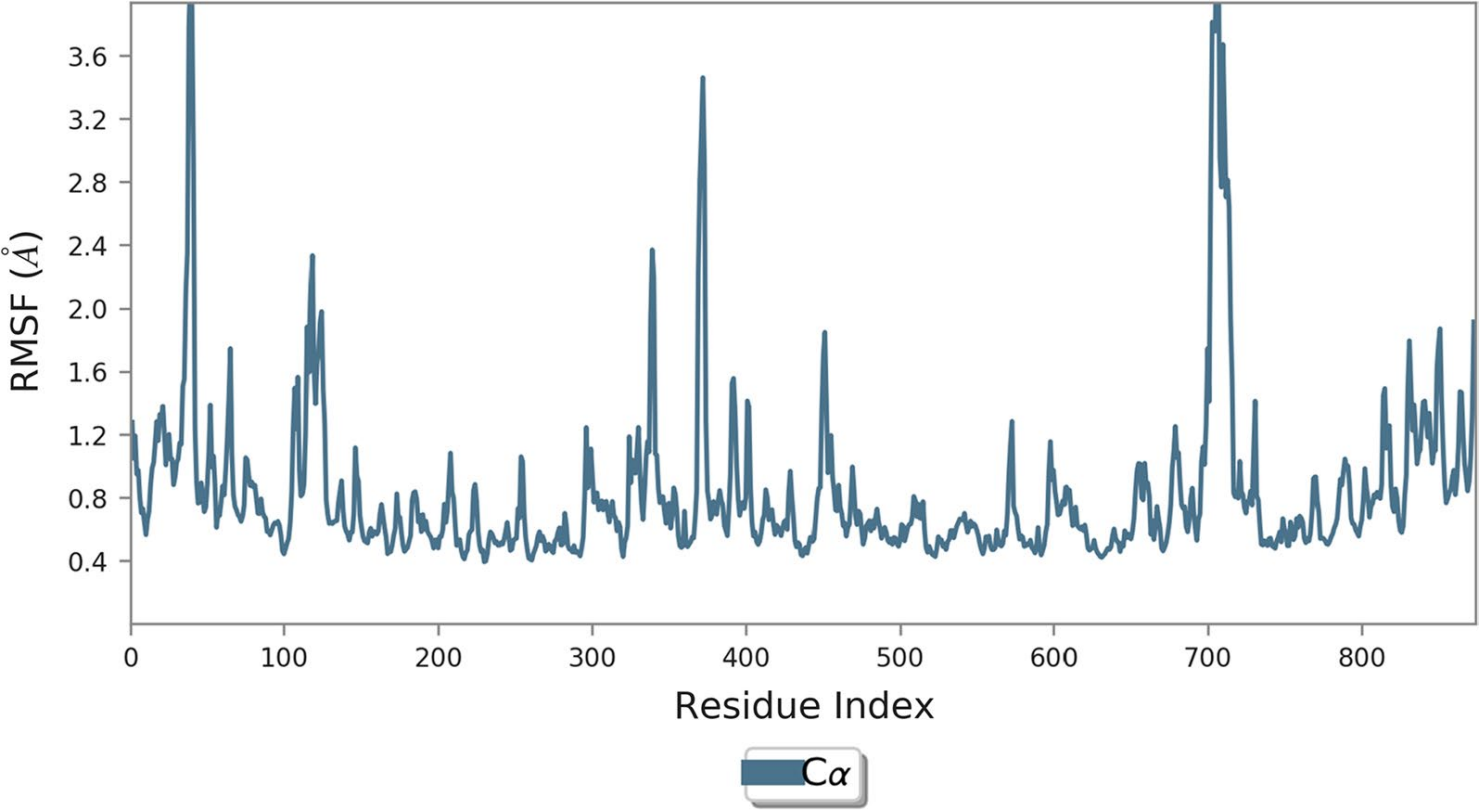
RMSF plot of the α-glucosidase residue in complexed with compound 1

The root mean square fluctuation (RMSF) is useful for characterizing local changes along the protein chain and the flexibility of the protein (Fig. 9).

**Fig. 10:**
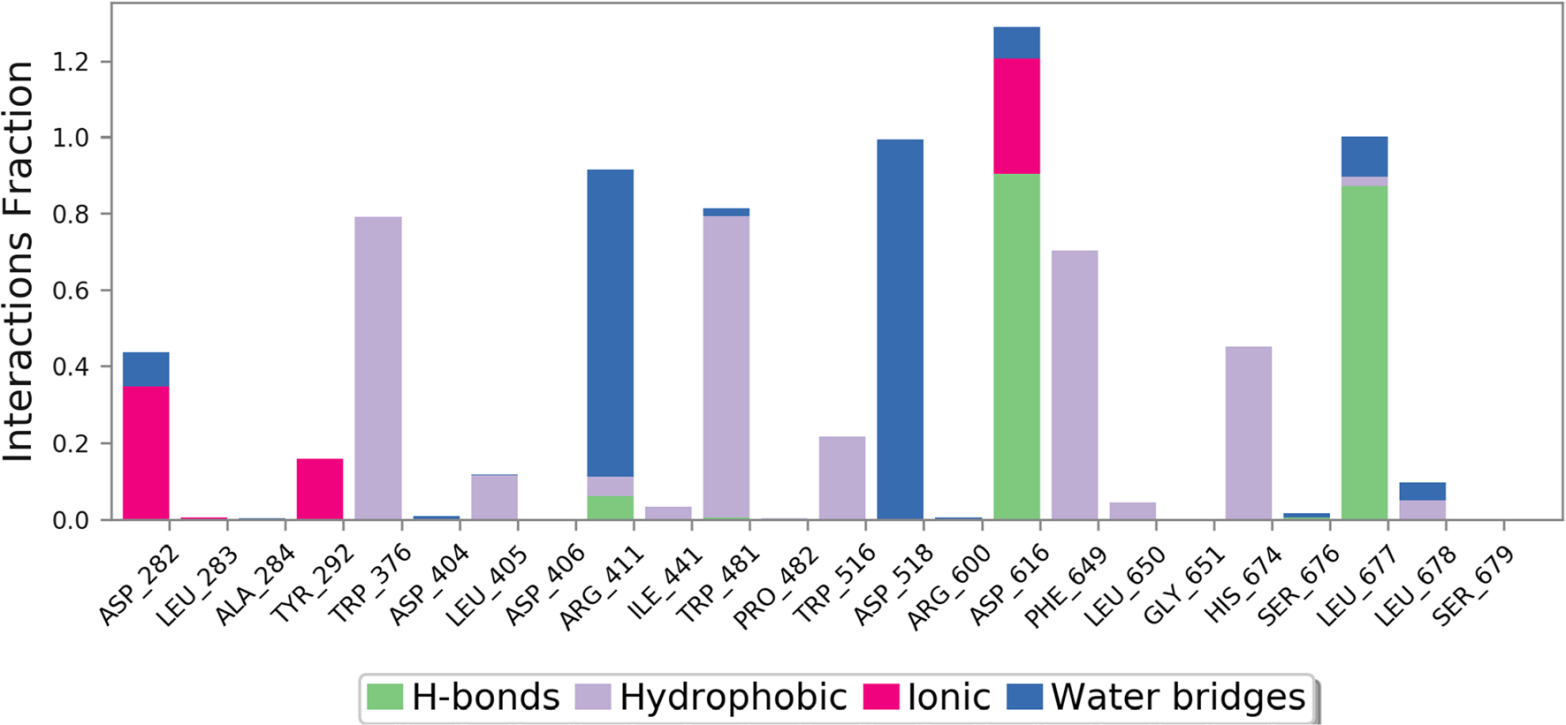
Protein–ligand contacts during the whole simulation time in α-glucosidase complexed with compound 1

Type and percent of protein interactions with the ligand throughout the simulation type are exhibited in Fig. 10. As can be seen interaction with Asp616 can be seen in almost 100% frame followed by Leu677, Asp518, Arg411, Trp376, and Phe649.

**Fig. 11:**
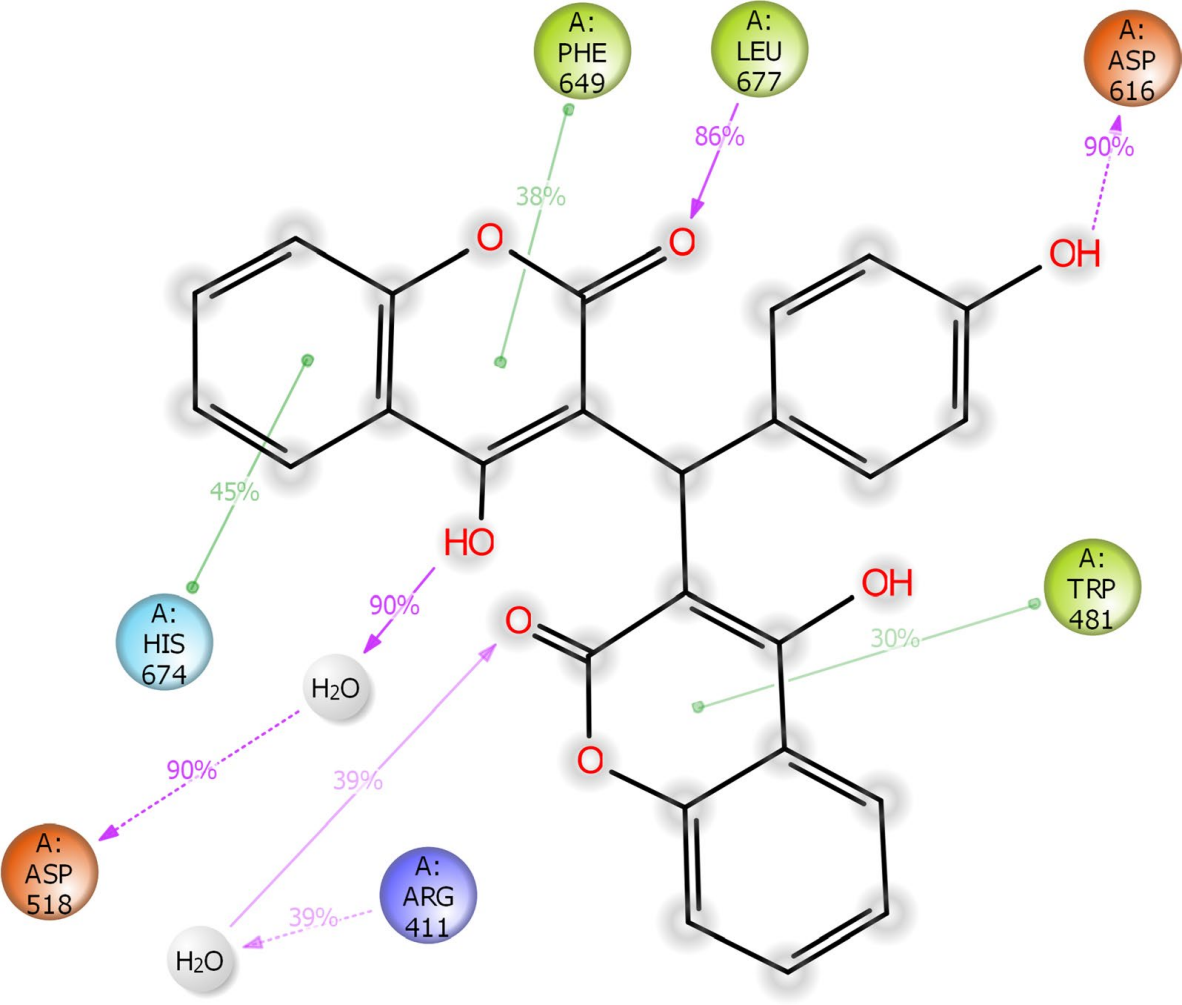
2D representation of ligand-residue interactions

Interactions that occur more than 30.0% of the simulation time in the selected trajectory (0.00 ns through 100.02 ns) are shown in Fig. 11. Asp616 participated in impotent hydrogen bond interaction with 4-hydroxy phenyl in more than 90% of cases. Also, Asp518 demonstrated interaction (90%) with OH of 2H-chromen-2-one through water bridge. Derivative 1 also exhibited interactions with Trp481, Phe649, Leu677, and His674. Another water bridge was also recorded between Arg411 and C=O of 2H-chromen-2-one (Fig. 11).

Overall, it can be seen that synthesized biscoumarin derivatives due to containing special functionality (OH and C=O and O) can participate in several interactions with the residue of the active site. Also, the planner structure of the aromatic ring in biscoumarin provides several pi-interactions with the active site which stabilizes the binding site of the enzyme. Also, the binding energy of compound 1 calculated using the MM/GBSA method is presented in Table 5.

**Table 5:** Binding free energy estimates via MM-GBSA after 100 ns MD simulations. All values are in Kcal/mol

Frame(ns)	ΔG_binding_	ΔG_gas_	ΔG_solv_	BOND	ANGLE	DIHED	VDWAALS	EEL	EGB	ESURF
− 26.664	− 36.924	10.26	0	0	0	− 33.9	− 3.024	14.628	− 4.368	− 3.64
− 31.776	− 45.96	14.184	0	0	0	− 39.516	− 6.444	18.828	− 4.644	− 3.87
− 19.452	− 32.592	13.128	0	0	0	− 26.976	− 5.616	16.332	− 3.192	− 2.66
− 18.744	− 32.004	13.26	0	0	0	− 26.988	− 5.028	16.476	− 3.216	− 2.68
− 17.16	− 29.076	11.928	0	0	0	− 25.128	− 3.948	14.988	− 3.072	− 2.56
− 33.108	− 42.156	9.048	0	0	0	− 40.044	− 2.112	13.74	− 4.692	− 3.91
− 30.552	− 39.636	9.084	0	0	0	− 38.112	− 1.536	13.596	− 4.512	− 3.76
− 29.04	− 38.352	9.312	0	0	0	− 36.78	− 1.572	13.74	− 4.428	− 3.69
− 28.116	− 42.636	9.048	0	0	0	− 36.048	− 6.588	18.888	− 4.38	− 3.65

*ΔG*_*binding*_*, ΔG*_*gas*_*, ΔG*_*solv*_  solvated binding free energy, binding free energy in a vacuum, solvation free energy, *BOND, ANGLE, DIHED*  bond length, bond angle, dihedral angle energies, *VDWAALS, EEL*  vdW interactions and electrostatics energies, *EGB, ESURF*   polar and nonpolar solvation energies

## Conclusion

In summary, an easy and efficient protocol for the synthesis of biscoumarin derivatives in the presence of MoO_3_ nanoparticles was explained. This methodology showed considerable synthetic advantages in terms of product diversity, simplicity of the reaction procedure, mild reaction conditions, and good to excellent yields. This procedure could be classified as green chemistry due to the elimination of any hazardous organic solvent. Similarity search analysis proposed the biscoumarin pharmacophore as ideal α-glucosidase inhibitors and molecular docking studies exhibited that these derivatives effectively fitted within the α-glucosidase active site with Chemscore value in the range of 17.56 to 29.49. MD assessments of compound 1 recorded critical interactions with Asp616, Leu677, Asp518, Arg411, Trp376, and Phe649 which prove the potential of this chemical family as promising drug candidates for further experimental analysis.

These findings demonstrated the applicability of similarity search followed by molecular docking and MD assessments as a promising method in the early stage of drug discovery.

## Data Availability

The datasets analyzed during the current study are available in the Worldwide Protein Data Bank (wwPDB) repository (https://www.rcsb.org/structure/5nn8).
